# Identification of Macrophage Extracellular Trap-Like Structures in Mammary Gland Adipose Tissue: A Preliminary Study

**DOI:** 10.3389/fimmu.2013.00067

**Published:** 2013-03-18

**Authors:** Sunish Mohanan, Sachi Horibata, John L. McElwee, Andrew J. Dannenberg, Scott A. Coonrod

**Affiliations:** ^1^Department of Biomedical Sciences, Baker Institute for Animal Health, Cornell UniversityIthaca, NY, USA; ^2^Department of Medicine, Weill Cornell Medical CollegeNew York, NY, USA

**Keywords:** peptidylarginine deiminases, PAD2, ETosis, macrophage extracellular traps, adipose tissue inflammation, histone citrullination, deimination, crown-like structures

## Abstract

PAD4-mediated hypercitrullination of histone H4 arginine 3 (H4R3) has been previously found to promote the formation of Neutrophil Extracellular Traps in inflamed tissues and the resulting histone H4 citrulline 3 (H4Cit3) modification is thought to play a key role in extracellular trap (ET) formation by promoting chromatin decondensation. In addition to neutrophils, macrophages have also recently been found to generate functional extracellular traps (METs). However, a role for PADs in ET formation in macrophages has not been previously described. Transcripts for PAD2 and PAD4 are found in mature macrophages and these cells can be induced to citrullinate proteins, thus raising the possibility that PADs may play a direct role in ET formation in macrophages via histone hypercitrullination. In breast and visceral white adipose tissue from obese patients, infiltrating macrophages are often seen to surround dead adipocytes forming characteristic “crown-like structures” (CLS) and the presence of these lesions is associated with increased levels of inflammatory mediators. In light of these observations, we have initiated studies to test whether PADs are expressed in CLS macrophages and whether these macrophages might form METs. Our preliminary findings show that PAD2 (and to a lesser extent, PAD4) is expressed in both in the macrophage cell line (RAW 264.7) and in CLS lesions. Additionally, we provide evidence that macrophage-derived extracellular histones are seen around presumptive macrophages within CLS lesions and that these histones contain the H4Cit3 modification. These initial findings support our hypothesis that obesity-induced adipose tissue inflammation promotes the formation of METs within CLS lesions via PAD-mediated histone hypercitrullination. Subsequent studies are underway to further validate these findings and to investigate the role in PAD-mediated MET formation in CLS function in the mammary gland.

## Introduction

ETosis is a recently described cell death-associated phenomenon that results in the release of a complex lattice of chromatin that contains DNA, histones, and other associated proteins (Amulic and Hayes, 2011; Liu et al., 2012; Saffarzadeh et al., 2012). These extracellular chromatin webs can be used to entrap and kill microbial organisms. Initially, this phenomenon was described in neutrophils, termed NETosis (Neutrophil Extracellular Traps), but subsequent studies have found that this mechanism also exists in other cell types such as macrophages, eosinophils, and mast cells. Given the ever-expanding array of cell types that can form extracellular traps (ET), this activity is now more generally referred to as ETosis. Various infectious agents such as bacteria, fungi, and protozoa, have been found to induce ETosis, as have cytokines, such as IL-8, and chemicals, such as phorbol myristate acetate (PMA) (Brinkmann et al., 2004).

At the molecular level, ETs were first shown to be comprised of decondensed DNA scaffolds and various neutrophil granular proteins (Brinkmann et al., 2004). Later proteomic studies further concluded that ETs contain histones (the most abundant fraction), elastase, glycolytic enzymes, and several other cytosolic proteins (Urban et al., 2009). Immunofluorescence (IF) studies have also shown additional components in ETs such as NADPH oxidase subunits, pentraxin-3, and cathelicidin. Interestingly, the localization of these proteins to NETs was not supported by subsequent biochemical studies, suggesting that some of these components may be loosely associated with ETs (Guimaraes-Costa et al., 2012). While the precise mechanisms underlying ET induction and formation are not completely understood, several morphological features of ETosis have been observed across various studies. These features include loss of distinct segregation of euchromatin and heterochromatin, disappearance of the lobular nuclear architecture in neutrophil nuclei, granular membrane disruption, and nuclear membrane swelling. ET induction is thought to occur when proteolytic enzymes are released from cytosolic granules where these enzymes then cause a collapse of the nuclear envelope, leading to disruption of plasma membrane integrity and, eventually, release of ETs into the extracellular space (Fuchs et al., 2007; Remijsen et al., 2011).

While many studies have now documented a role for NETs in an array of activities, much less is known about ETosis in other cell lines such as macrophages. Two recent studies found that the bacterial organisms *Histophilus somni* and *Mannheimia haemolytica* can induce ETs in bovine macrophages (METs) (Aulik et al., 2012; Hellenbrand et al., 2013). Additionally, another study showed that human THP-1-derived macrophages and the RAW 264.7 macrophage cell line formed METs in response to *E. coli* toxins. Interestingly, while MET formation has been documented in tissue macrophages, it has yet to be reported in peripheral blood monocytes (Aulik et al., 2012).

PAD enzymes catalyze the conversion of positively charged arginine residues to neutrally charged citrulline in a hydrolytic reaction termed citrullination or deamination. The resulting loss of charge at this site can dramatically alter the target protein’s tertiary structure as well as its ability to interact with other proteins (Wang et al., 2009; Mohanan et al., 2012). The N-terminal tails of histones such as H3 and H4 are arginine-rich and appear to represent major target for PAD enzymes. For example, numerous reports have shown that PAD4 and, more recently, PAD2, regulate gene expression via citrullination of histone H4R3 and H3R26, respectively (Wang et al., 2009; Cherrington et al., 2012). While the mechanisms by which histone citrullination regulates gene transcription are not fully understood, we recently demonstrated that PAD2-catalyzed histone citrullination promoted localized chromatin decondensation at target gene promoters, thus likely facilitating binding of the basal transcriptional machinery (Zhang et al., 2012). On a more global level, we have also recently shown that activation of PAD4 in neutrophils promotes histone hypercitrullination, global chromatin decondensation, and NET formation (Wang et al., 2009). In this previous study, we showed by transmission electron microscopy that activation of PAD4 in HL60 granulocytes promoted the conversion of multi-lobular heterochromatic nuclei into a more round euchromatic nuclear pattern (Wang et al., 2009). Additionally, we demonstrated that TNF-α treatment of blood neutrophils resulted in the release of extracellular chromatin that was extensively citrullinated at histone H4R3. The link between the H4Cit3 modification and NET formation is very strong and this modification is now routinely utilized to document the presence of ETs in cells and tissues (Neeli et al., 2008; Wang et al., 2009). In addition to TNF-α, LPS and H_2_O_2_ have also been shown to induce PAD-mediated histone deimination (Neeli et al., 2008). Importantly, the requirement of citrullination in NET formation *in vivo* was recently documented by investigators who showed that PAD4^−/−^ mice have reduced ability to form NETs in response to various stimuli. Additionally, the investigators found that these mice are more susceptible to bacterial infections (Li et al., 2010). More generally, PAD activity is also closely associated with non-microbial induced immune-mediated inflammatory activity such as that seen in autoimmune arthritis, colitis, and chronic obstructive pulmonary disease. Along these lines, excessive PAD-mediated ETosis has also recently been found to play a role in deep vein thrombosis and cystic fibrosis (Marcos et al., 2010).

Obesity is a major public health concern and, among other things, is a risk factor for hormone receptor-positive breast cancer in postmenopausal women (Cleary and Grossmann, 2009; van Kruijsdijk et al., 2009). Numerous studies in obese humans and animals have shown that macrophages infiltrate visceral adipose tissue and surround dead adipocytes forming a characteristic “crown-like structure” (CLS) morphology. These macrophages are believed to primarily function at these sites by “cleaning up” the remnants of dead/dying adipocytes via phagocytosis of lipids, cytoplasmic debris, and karyorrhectic remnants (Cinti et al., 2005). Additionally, these immune cells produce proinflammatory mediators that are found in the circulation of obese women and have been linked to breast cancer progression.

Recent studies have shown that CLS lesions occur in white adipose tissue of the breast in obese women and in the mammary gland of experimental models of obesity (Morris et al., 2011; Subbaramaiah et al., 2011). Importantly, in mouse models of obesity, CLS occur in association with activation of NF-κB and elevated levels of inflammatory mediators including TNF-α, IL-1β, and COX-2 (Subbaramaiah et al., 2012). Increased levels of TNF-α at these sites is thought to enhance inflammatory activity due to a paracrine signaling loop between TNF-α, saturated fatty acids, and adipocytes (Suganami and Ogawa, 2010). Given that TNF-α can induce ET formation in neutrophils, that PADs are intimately associated with inflammation, and that macrophages can form ETs, we predicted that macrophages may undergo ETosis in CLS lesions. As outlined below, we performed a preliminary study to test this hypothesis. Our findings suggest that METs do form in CLS structures, thus laying the groundwork for future studies to investigate the functional role for METs in the resolution of inflamed white adipose tissue in the mammary gland.

## Materials and Methods

### RAW 264.7 cell culture and MET induction

RAW 264.7 mouse macrophage cells were grown in RPMI 1640 (Cellgro, Manassas, VA, USA) supplemented with 10% fetal bovine serum containing penicillin-streptomycin. The cells were grown in a sterile, humidified incubator, and maintained in these conditions at 37°C and 5% CO_2_. To perform IF staining, macrophages were grown on 12 mm glass coverslips for 2 days at 37°C (Fisher Scientific, Hanover Park, IL, USA). The RAW 264.7 macrophages were treated with recombinant TNF-α (20 ng/ml, R&D systems, Minneapolis, MN, USA) for 2 h, then washed in 1× phosphate buffered saline (PBS), fixed in 4% cold paraformaldehyde for 20 min, washed in 1× PBS, and IF staining was performed as described below.

### Mammary gland adipose tissue from obese mice

Mammary gland adipose tissue sections were prepared from a dietary model of obesity as described previously (Hong et al., 2009; Subbaramaiah et al., 2011). Ovariectomized C57BL/6J mice (Jackson Laboratories) at 5 weeks of age were given high fat diet (60 kcal% fat, D12492i, Research Diets) to generate obese mice. Mice were fed *ad libitum* for 10 weeks and sacrificed to collect mammary gland tissue. Tissue samples were formalin fixed for histological and immunohistochemical analyses. The animal protocol was approved by the Institutional Animal Care and Use Committee at Weill Cornell Medical College.

### mRNA isolation and RT-PCR

RNA was isolated from the RAW 264.7 macrophage cells using the Qiagen RNeasy mini kit, including on-column DNAse treatment to remove genomic DNA (Qiagen # 74104). The purified RNA was reverse-transcribed using the Applied Biosystems High Capacity RNA-to-cDNA kit according to manufacturer’s protocol (Applied Biosystems # 4387406, Foster City, CA, USA). The cDNA (100 ng) was mixed with Go-Taq DNA polymerase (Promega # M3005, Madison, WI, USA) and primer set. The following primer pairs were used (size of the amplicons are in parentheses): PAD2 primer set #1 (400 bp): Fwd – 5′-AGAAGGGAGGCTCTGAGGTC-3′ and Rev – 5′-CTGGCCAGAGAATTGAGGAC-3′; PAD2 primer set #2 (188 bp): Fwd – 5′-CAAGATCCTGTCCAATGAGAG-3′ and Rev – 5′-ATCATGTTCACCATGTTAGGGA-3′; PAD4 primer set #1 (406 bp): Fwd – 5′-TCTCCCTGCTGGACAAGTCT-3′ and Rev – 5′-AGCCCAGTGAGCTCTGACAT-3′; PAD4 primer set #2 (193 bp): Fwd – 5′-CTACTCTGACCAAGAAAGCC-3′ and Rev – 5′-ATTTGGACCCATAACTCGCT-3′; GAPDH primer set #1 (324 bp): Fwd – 5′-CCCACTAACATCAAATGGGG-3′ and Rev – 5′-ATCCACAGTCTTCTGGGTGG-3′; and GAPDH primer set #2 (209 bp): Fwd – 5′-GGGCATCTTGGGCTACAC-3′ and Rev – 5′-GGTCCAGGGTTTCTTACTCC-3′. PCR was performed using the following set up: 5 min at 95°C, 30 cycles of (30 s, 94°C; 30 s, 58°C; 30 s, 72°C), 5 min at 72°C. The PCR products were analyzed on a 1% agarose gel using Geldoc (BioRad) software.

### Immunohistochemistry and immunofluorescence

Immunohistochemistry (IHC) and IF experiments followed our previously described protocol (Cherrington et al., 2010). Briefly, paraffin embedded tissue sections were deparaffinized and rehydrated in 3 × 5 min washes in xylene followed by single sequential 5 min washes in 100, 95, and 75% EtOH. Slides were then incubated for 10 min in 0.5% hydrogen peroxide in methanol to quench endogenous peroxidases. Antigen retrieval was performed by boiling slides 2× for 10 min in 0.01 M sodium citrate pH 6.8 and, after cooling, slides were washed in 1× PBS. Tissue slides and fixed coverslips containing RAW264.7 cells were then blocked in 10% normal goat serum and 2× casein (Vector Labs, Burlingame, CA, USA) for 20 min at room temperature in a humidified microprobe chamber. Slides were blotted to remove excess blocking solution and then primary antibody diluted in 1× PBS was applied to the slides for 2 h at room temperature. Slides or cover slips were then stained with either anti-PAD2 (ProteinTech #122100-1-AP, Chicago, IL, USA) at a 1:100 dilution, anti-PAD4 (Sigma-Aldrich # P4749) at a 1:100 dilution, or with anti-histone H4 Citrulline 3 (Millipore-Upstate # 07-596) at a 1:100 dilution. Following 3× washing with 1× PBS, slides were incubated with a 1:200 dilution of biotinylated secondary antibody (in 1× PBS) for 1 h at room temperature and then washed 3× in 1× PBS. For IHC, slides were incubated in DAB chromagen (Vector Labs) solutions according to the manufacturer’s protocol, washed and then counterstained with Gill’s hematoxylin, and coverslipped. For each experiment, duplicate slides were treated with control rabbit IgG antibody at the appropriate concentration as a negative control. For immunofluorescence, slides were incubated in streptavidin conjugated-488 or 555 (Invitrogen), washed, and then mounted using Vectashield containing DAPI (Vector Labs). As a negative control, duplicate slides were treated with control rabbit IgG at the appropriate concentrations.

In order to quantitate changes in levels of citrullinated histones following TNF-α treatment, a time course experiment was performed evaluating H4Cit3 immunofluorescence intensity at 0, 2, 6, and 24 h. RAW 264.7 macrophages were treated with 20 ng/ml of TNF-α, fixed, and H4Cit3 immunofluorescence staining was performed using the method described above. Five different fields from each time point were imaged using confocal microscopy and the mean integrated fluorescence intensity was calculated using ImageJ software analysis as previously described by Gavet and Pines (2010). The results were then analyzed by one-way ANOVA to detect statistical differences and a *p*-value < 0.01 was considered significant. All values are presented as mean ± SD.

## Results and Discussion

### TNF-α appears to induce MET formation in RAW 264.7 macrophages

**Figure 1:**
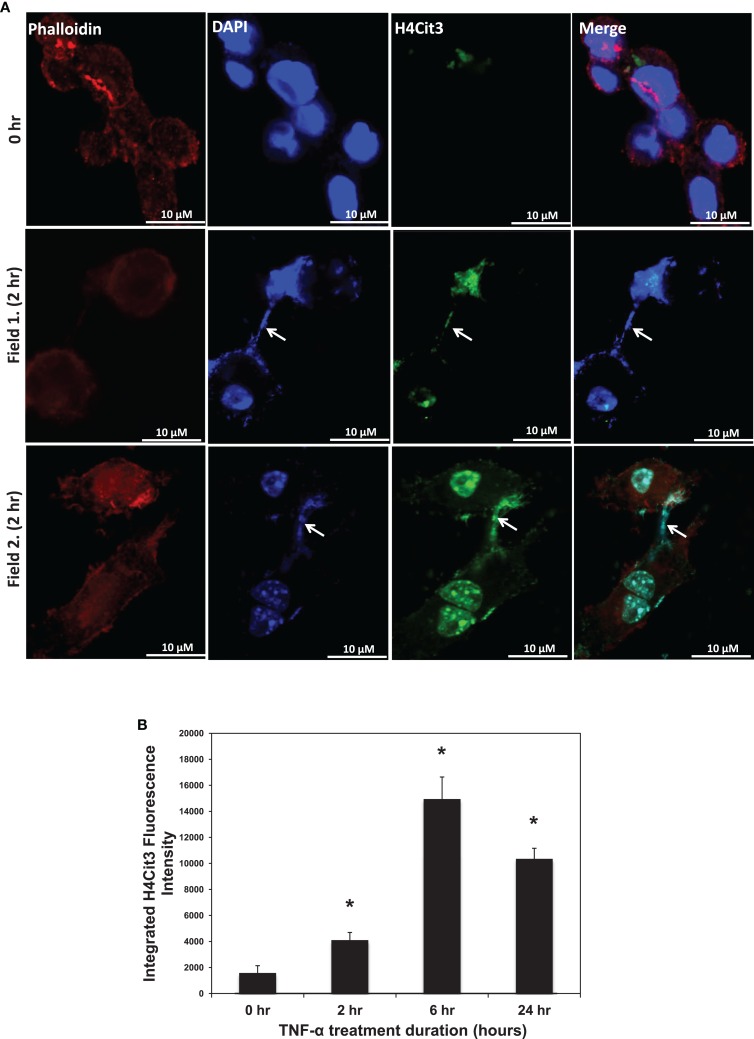
**(A)** H4Cit3 immunofluorescence staining in RAW 264.7 macrophages following 0 and 2 h TNF-α treatment. Cultured RAW 264.7 macrophage cells appear to form METs following TNF-α stimulation and these METs contain citrullinated histones. DAPI staining (blue) shows that DNA extends beyond the Phalloidin-stained cell borders. Anti-Histone H4Cit3 staining shows presence of citrullinated histones within METs (Arrows). Two separate fields from the 2 h treatment group (Fields 1 and 2) are included for reference. **(B)** Integrated anti-H4Cit3 fluorescence intensity quantitation at 0, 2, 6, and 24 h of TNF-α treatment. The RAW 264.7 macrophages were treated with 20 ng/ml of TNF-α, fixed, stained with the anti-H4Cit3 antibody, and imaged by indirect immunofluorescence at the indicated time points. Five unique fields were imaged by confocal microscopy at each time point and the mean integrated fluorescence intensity was calculated using ImageJ software analysis. Results were analyzed by ANOVA and the graphs represent mean ± standard deviation (*p-value < 0.01).

Given that TNF-α induces ET formation in neutrophils, we first tested whether TNF-α could promote ET formation in the RAW 264.7 macrophage cell line. As shown in Figure 1A, we found that TNF-α treatment led to DAPI-stained chromatin in characteristic strands outside of ∼5–10% of the phalloidin-bound RAW264.7 cells. Two different microscopic fields from 2 h treatment groups are shown in Figure 1 as representative images. By contrast, these characteristic strands were not seen in untreated control cells. This observation supports the hypothesis that TNF-α induced the release of chromatin from the cell nucleus into the extracellular space. As a further test of this hypothesis, we then stained the TNF-α-treated RAW264.7 cells with the anti-H4Cit3 antibody. Results show that the extracellular chromatin appeared to be extensively citrullinated at H4R3, thus suggesting that PAD-mediated histone hypercitrullination promotes ETosis in macrophages. In an effort to quantitate the extent to which TNF-α promoted the formation of extracellular chromatin in RAW264.7 cells, we quantitated the staining intensity for the anti-H4Cit3 antibody at different time points following treatment. Results show that the mean integrated fluorescence intensity at 2, 6, and 24 h was significantly increased (as determined by ANOVA) when compared to the 0 h time point (*p* < 0.01). We also note that the H4Cit3 intensity gradually increased until the 6 h time point, while levels were lower at the 24 h time point. This result supports the hypothesis that, as with neutrophils, TNF-α stimulates histone hypercitrullination and ETosis in macrophages.

### PAD2 is a likely candidate for catalyzing histone hypercitrullination during ETosis in macrophages

**Figure 2:**
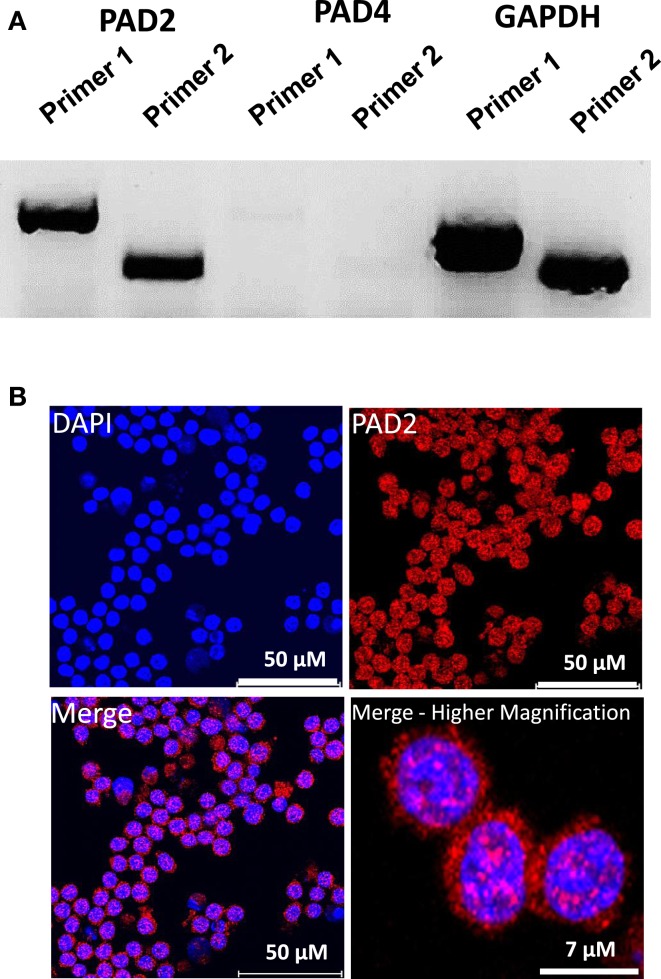
**PAD2 expression in cultured RAW 264.7 macrophage cells**. **(A)** RT-PCR for PAD2 and PAD4 mRNA expression in cultured RAW 264.7 macrophages. Two unique primer sequence sets were used to test for PAD2 and PAD4 mRNA expression levels. RAW 264.7 macrophages were found to strongly express PAD2 mRNA. GAPDH mRNA expression levels were used as the internal control for RT-PCR. **(B)** Anti-PAD2 immunofluorescence labeling shows positive staining in the nucleus and cytoplasm of RAW 264.7 macrophages. Nuclei are stained with DAPI.

While PAD4 is the only PAD family member to have been shown to be required for ETosis, both PAD2 and PAD4 have been previously shown to be expressed in macrophages. Therefore, as an initial investigation into which PAD family member may catalyze ET production in RAW264.7 cells, we first investigated mRNA expression levels of PAD2 and PAD4 in this cell line. Surprisingly, RT-PCR analysis found that PAD4 expression was very low in RAW264.7 cells (Figure 2A), suggesting that this family member may not play a critical role in macrophage function. We note, however, that we have yet to test for PAD4 levels in *in vivo*-derived macrophages. Given the high level of PAD2 mRNA observed in these cells, we then carried out indirect immunofluorescence to test whether RAW264.7 macrophages also expressed PAD2 protein. Results showed PAD2 staining was strong in these cells (Figure 2B) and appeared to localize, in part, to DAPI-poor euchromatic regions of the RAW 264.7 cell nucleus (Figure 2B, bottom right panel). While not conclusive, this finding supports the hypothesis that PAD2 is a primary driver of histone hypercitrullination, chromatin decondensation, and ET formation in macrophages. We note that the localization of PAD2 to the cell nucleus fits well with this hypothesis. Additionally, this hypothesis is further supported by our recent finding that both global and promoter-specific chromatin decondensation is catalyzed by PAD2 in breast cancer cell lines (Zhang et al., 2012).

### Evidence supporting the hypothesis that METs exist in mouse mammary gland CLS lesions

**Figure 3:**
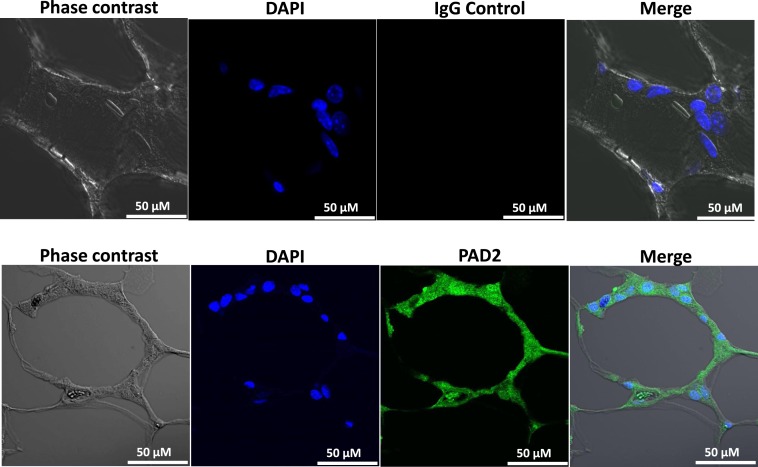
**PAD2 expression in cells within CLS lesions of the murine mammary gland**. Immunofluorescence staining of mammary gland adipose tissue sections with anti-PAD2 antibodies shows that cells within the CLS lesions appear to express PAD2. Nuclei are stained with DAPI. Non-specific rabbit IgG was used as a negative control.

**Figure 4:**
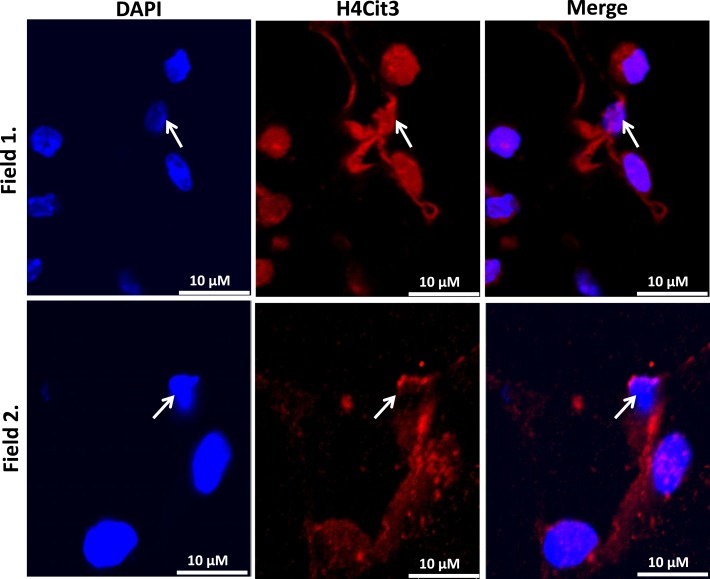
**Extracellular trap-like structures within CLS lesions of the murine mammary gland stain positive for the histone H4Cit3 modification**. Immunostaining of mouse mammary gland adipose tissue with the anti-H4Cit3 antibody shows that extranuclear citrullinated histones appear to extend into the extracellular space between cells within CLS lesions. Arrow highlights nuclei from which the extracellular histones appear to originate.

**Figure 5:**
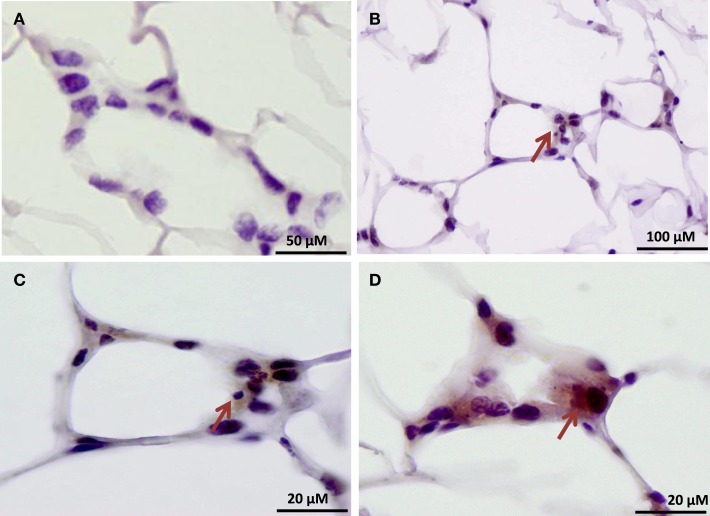
**Immunohistochemical localization of the histone H4Cit3 modification within CLS lesions of the mammary gland**. **(A)** Negative control showing adipose tissue sections stained with non-specific rabbit IgG and hematoxylin counterstain. **(B–D)** Arrows indicate CLS lesions with positive immunostaining for the H4Cit3 modification. A higher magnification of the CLS lesion from **(B)** is given in **(C)**.

Our findings in the RAW264.7 cell line suggested that PAD2-catalyzed histone hypercitrullination may promote ETosis in CLS-localized macrophages. In order to begin testing this hypothesis, we next investigated whether PAD2 was expressed in the nucleus of macrophages in CLS lesions. Results show that PAD2 expression appeared to be robust in these cells (Figure 3). Importantly, similar to the RAW264.7 cells, PAD2 was found to concentrate in the DAPI-poor regions of the presumptive macrophage cell nucleus, again suggesting that PAD2 localizes to euchromatic regions of the nucleus. We then stained these samples with the anti-H4Cit3 antibody and found positive staining for the histone H4Cit3 modification extending from numerous CLS cell nuclei (Figure 4). This staining is highlighted by arrows in the two representative images (Figure 4, Field 1 and Field 2) and appears to extend into the extracellular space between CLS cells. In order to more precisely refine the localization of H4Cit3-stained chromatin, we next carried out immunohistochemical staining of the CLS-containing sections using the anti-histone H4Cit3 antibody. Results showed that CLS cell nuclei were frequently stained with this antibody (Figure 5). Importantly, the anti-H4Cit3 staining is particularly intense in fragmented nuclear particles and, in such cases, the staining appeared to extend well into the cell cytoplasm and extracellular space.

**Figure 6:**
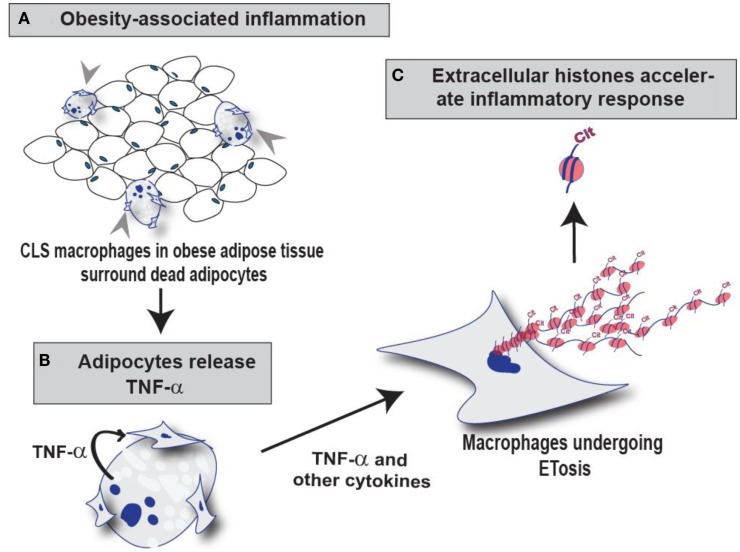
**Schematic illustration of the role of PAD2-mediated histone citrullination during MET formation in CLS lesions**. **(A)** Macrophages infiltrate obese adipose tissue and surround dead adipocytes forming the CLS lesions. **(B)** TNF-α and other cytokines released from adipocytes may stimulate ETosis in these surrounding CLS macrophages thus promoting PAD2-mediated histone citrullination, chromatin decondensation, and extracellular chromatin scaffold formation. **(C)** We hypothesize that the citrullinated histones released during MET formation may regulate the inflammatory microenvironment of mammary adipose tissue by influencing the macrophage infiltration and/or macrophage activation.

Outcomes from this study suggest that macrophages undergo ETosis following TNF-α stimulation *in vitro* and within mammary gland CLS lesions in obese mice. Our IF and IHC experiments using the anti-H4Cit3 antibody (a well validated marker for ET chromatin) finds that histone hypercitrullination is seen in extracellular chromatin in both RAW264.7 cells and in CLS macrophages, thus supporting the hypothesis that the observed ETosis is likely PAD-mediated. Additionally, we show that PAD2, but not PAD4, expression is robust within the nucleus of RAW264.7 cells and CLS-localized macrophages, thus identifying PAD2 as a strong candidate for catalyzing ETosis formation in macrophages. These preliminary findings provide support for the hypothesis that METs occur in CLS lesions of mammary gland adipose tissue from obese mice and lay the groundwork for future studies aimed at identifying a role for METs in CLS function. Given the close ties between PAD activity and inflammation, we favor the hypothesis that the release of hypercitrullinated histones from the macrophage nucleus into the extracellular space during MET formation plays a critical role in promoting inflammatory signaling pathways within microenvironment of mammary gland adipose tissue (Figure 6). Experiments are currently underway to test this hypothesis.

## Conflict of Interest Statement

The authors declare that the research was conducted in the absence of any commercial or financial relationships that could be construed as a potential conflict of interest.
